# Late-Stage Capitalism and the Canadian Polycrisis in Living and Working Conditions: Implications for Health and Means of Responding

**DOI:** 10.1177/27551938251411280

**Published:** 2026-01-06

**Authors:** Rozhin Amin, Dennis Raphael

**Affiliations:** 1 PhD Program in Health Policy and Equity, York University, Toronto, Ontario, Canada; 2 School of Health Policy and Management, 7991 York University, Toronto, Ontario, Canada

**Keywords:** health equity, polycrisis, social determinants of health, capitalism, neoliberalism

## Abstract

There is growing concern about the health and overall well-being of societies stemming from neoliberal-oriented governments reducing their management of the economy, weakening programs and supports for the population, and shifting public goods to the private sector. As a result, a polycrisis exists in many nations related to various key social determinants of health. In this paper, we argue the Canadian polycrisis is due to the contradictions within Canadian society between the economic and political imperatives of capital accumulation (ie, profit making) with social reproduction (ie, societal continuity) associated with late-stage global capitalism. These contradictions threaten societal functioning: declining redistribution of income and wealth, reduced social spending, unwillingness to manage the market economy, and unrelenting privatization of activities once part of the public sphere. The result has been a Canadian polycrisis of growing food and housing insecurity, precarious employment, widening income and wealth inequalities, and a healthcare crisis. We argue responding to the polycrisis requires recognizing and dealing with the contradictions generated by neoliberal capitalism through profound reform or even transformation of the economic system toward a post-capitalist, socialist economy. We consider how such reforms or transformations can come about.

## Introduction

Since the mid-1970s there has been a global increase in the sense of uncertainty in people's lives.^1–3^ The contemporary period is marked by social, geopolitical, ecological, and economic crises that rather than being isolated, represent an enduring and interconnected condition known as a polycrisis.^4^ For the Canadian Labour Congress, these challenges include escalating prices for essential goods, persistent high inflation paired with stagnating wages, and overburdened health and social care systems.^5^ In such an environment, people are seeking ways to escape this dystopia by identifying alternative ways of organizing society.^1,3,6^

For many, the polycrisis is simply a result of various converging issues that can be dealt with through adjustments to public policy. For others, including ourselves, the polycrisis is the result of the contemporary capitalist social order, specifically, the shift to neoliberalism that began in many wealthy democracies in the late 1970s.^1,6–8^

Historically, these adverse effects generated various efforts to counter the effects of capitalist organization of societies. In the nineteenth century, the harmful effects of the industrial revolution in England led German and Austrian authorities to develop public healthcare and public pensions.^9^ The early twentieth century saw similar developments in Scandinavia.^10^ These approaches to governance—now known as welfare regimes—manage the social relationships between governing authorities, capitalist entities, and workers, thereby ensuring both capital accumulation and social reproduction. More recently, an additional entity—civil society—has entered this mix.^11^

All welfare regimes reached their apex in the post-World War II period (1945-1975) as governing authorities accepted the Keynesian view that since economic and social instability would lower demand and harm economic growth, states should expand their involvement in economic and social affairs. The result was the Golden Age of Capitalism—that included increased provision of health and social services, encouraged income increases across all social classes, and made the welfare state a central feature of all wealthy nations.^12^

By the end of the 1960s, the Keynesian framework began to unravel. The global stagflation of the 1970s—largely attributed to the oil crisis—threatened capital accumulation. As states faced declining tax revenues and rising social expenditures, free-market economists argued for the necessity of neoliberalism as a model of governance.^13^ Others, however, saw these arguments as a power grab by the corporate and business sector to increase profits and roll back the economic and social reforms of the post-WWII era.^12,14^

As a result, most welfare regimes were restructured during the 1980s along neoliberal lines, reshaping the role of the state by reducing its management of the economy, lowering social spending and encouraging market-based solutions to social problems. While all wealthy nations faced the pressures of global neoliberalism, responses differed according to domestic politics such that the Anglo-Saxon liberal welfare states of Canada, USA, and UK were especially susceptible to such influences.^15,16^ Market-driven income inequality increased in Canada during this period, and public policies inspired by the neoliberal siren saw massive corporate income tax cuts and reduced social spending that led to declining quality and greater skewing of the distribution of living and working conditions.^17^ Since these conditions are also social determinants of health (SDOH), population health stagnated such that Canadian life expectancy at birth declined in rank against many other Organization for Economic Cooperation and Development (OECD) countries from fifth in 1991 to 17th in 2021.^18^ In terms of infant mortality, Canada's performance has also stagnated. In 1991, Canada was ranked ninth in infant mortality. It is now ranked 30th of 36 OECD nations.^19^

Existing literature considers how these neoliberal-inspired shifts in public policy have shaped the quality and distribution of important SDOH, but these shifts have not been explicitly linked to the ongoing contradictions between the imperative of capital accumulation in capitalist economies and the requirements for social reproduction.^20^ This article addresses this conceptual gap by applying a critical materialist political economy approach (CMPE) to interrogate the growing contradictions between capital accumulation and social reproduction in Canada and its effects upon the quality and distribution of the SDOH of income, employment security and working conditions, housing, food security, and healthcare—thereby creating the polycrisis. We then propose alternative futures to our current dystopia. In this article we examine how Canada's liberal welfare state under neoliberal pressures increasingly supports capital accumulation at the expense of social reproduction leading to a decline in the quality and equitable distribution of the SDOH for many Canadians. We see these processes as part and parcel of capitalism's latest manifestation and argue these features require either radical reform or transformation of this economic system.

## Methodology and Method

We apply a CMPE—informed by critical realism—lens to analyze how contemporary public policy initiatives in Canada represent the contradiction between capital accumulation and social reproduction. CMPE places these contradictions within the imperatives of contemporary global capitalism and critical realist analysis (CRA) directs attention to the oppressive social structures and the power dynamics that sustain them through a four-stage approach.^21^ The first stage identifies problems such as unmet needs, suffering, or false beliefs. The second identifies the sources or causes of these problems by linking them to specific forms of domination or power structures perpetuating these issues. The third is a critical evaluation that recognizes their detrimental impact. The final stage identifies actions that will remove these sources of oppression.^22^

The concept of polycrisis characterizes an array of linked crises and how these crises are not simply contemporaneous but also interconnected. In such a situation, conventional public policy management methods are likely to be ineffective as the polycrisis extends beyond the reach of any single policy area but reflects the overall structures and processes of a society's political economy.^1^ The depth of the polycrisis in Canada is illustrated in a recent analysis documenting the health threatening similarities between Victorian-era capitalist practices in the mid 1850s and contemporary Canadian capitalism in income inequality, employment security and working conditions, and access to food, housing, and health services.^23^

We see the polycrisis—with its adverse effects upon numerous social determinants of health—as embedded in the ideological and political framework of Canada's form of capitalism that shapes the current policy environment. Neoliberal-informed public policies that promote capital accumulation at the expense of social reproduction worsen the working and living conditions of many Canadians, thereby creating pervasive insecurity. How extensive is this uncertainty? IPSOS reports 62% of Canadians believe that the country is on the wrong track and over 70% believe government and public services will soon be inadequate.^24^ This widespread uncertainty is driving interest in envisioning post-capitalist imaginaries whereby societies are structured on principles other than those common to capitalism.^23^

Capitalism requires society be organized along profitmaking or capital accumulation by the production and then selling of products at values greater than what was involved in its production.^25^ These processes of production and distribution must be legitimized as these arrangements usually produce profound differences in life chances which have come to be called social inequalities. Braudel notes that “Capitalism, the privilege of the few, is unthinkable without the active participation of society.” Legitimization requires the consent of those being governed, which if not attained, can escalate into organized resistance.^26^

Capitalism transformed the world, undergoing substantial changes itself in the process. Ross and Trachte place modern capitalism's evolution into three distinct eras which remain relevant 35 years after their formulation: entrepreneurial capitalism, monopoly capitalism, and global capitalism.^16^ Their main contribution was identifying the phase of capitalism that began in 1975: global capitalism which emphasized the free movement of capital across borders, with multinational corporations operating on a global scale and influencing global trade, production, and labor markets. Ross and Trachte view this phase as dominated by corporate interests, where the demands of the working class are marginalized, public expenditure on supports and services is cut back, and there is a stagnation in the purchasing power of the working class. Its effects are now seen as the polycrisis of rising income and wealth inequalities, precarious employment, housing unaffordability, growing food insecurity, and declining access to healthcare.^26,27^ The Canadian welfare state, designed to manage these by-products of capitalism is proving increasingly ineffective in achieving this goal.

Esping-Andersen categorizes Canada as a liberal welfare state fundamentally grounded in the ideology of liberty whereby the market is assumed to provide all with their basic needs.^10^ In practice, its prioritizing of market solutions fails to accomplish this goal. Between its healthcare system and its overall approach to economic and social security, Canada's welfare state is more generous than the USA, but shows greater similarity to the USA version than to the more developed European welfare states. In fact, Canada has received numerous rebukes from the United Nations for its failure to provide its citizens with economic and social security, particularly in the areas of income, housing, food security, and treatment of women, Indigenous peoples, and persons with disabilities.^17^

In addition to Canada's being a liberal welfare state, its making of public policy has been subjected since the mid-1970s to governments’ acceptance of the ideology of neoliberalism and the imposition of austerity measures.^28,29^ This shift in public policy has favored welfare state retrenchment and government abdication of responsibility for the welfare of Canadians. Its effects are seen across a wide range of policy domains.^30^

Our analysis is presented as a narrative research review of scholarly articles and government reports that consider the Canadian public policy scene within the context of late-stage capitalism and its neoliberal approach to governance. We used Google Scholar™ to search the terms “public policy,” “Canada,” “social determinants of health,” “neoliberalism,” “capitalism,” “employment security, working conditions, food insecurity,” “income,” “housing,” “healthcare,” and “late-stage capitalism.” Google Scholar™ identifies significantly more citations than the WoS Core Collection and Scopus across all subject areas.^31^

## Findings

### Income and Wealth Inequality

Over recent decades, neoliberal theoreticians argued globalization and economic growth would boost living standards for all. However, in Canada, the benefits have been heavily concentrated among the wealthiest individuals. By 2021, the top quintile increased their share of market income to 52.6% of all market income indicating an increase from 44.7% in 1980.^32^

This same quintile in 2021 controlled 67.1% of wealth while the lowest two quintile of Canadians held only 2.8%.^33^ As a result, market income inequality in Canada rose from a Gini index of 0.37 in 1980 to 0.42 in 2023. Growing market income inequality was partially mitigated during the 1980s by transfers and taxes, which reduced market income inequality by 0.023 points on the Gini index. However, in the 1990s, as Canada shifted toward neoliberal policies, redistribution measures only offset 0.014 points. By the 2000–2010 period, these policies made no measurable progress in reducing inequality. Consequently, after-tax income inequality in Canada increased from a Gini index of 0.28 in 1980 to 0.30 in 2023.^34,35^

### Employment and Working Conditions

The OECD defines low pay as earnings that are less than two-thirds of the median hourly or annual earnings for all full-time workers. Using this definition, in 2022, 19% of full-time workers in Canada were classified as being in low-wage employment. This places Canada 32nd among the 38 OECD countries, surpassed only by the USA, the UK, and the Eastern European countries of Latvia, Lithuania, and Slovenia.^36^ Low pay is a result of the weak state of the organized labor sector.

Over the past few decades, the persistent application of neoliberal principles across Canada created an environment that discourages worker militancy, exacerbates worker insecurity, encourages wage restraint, and weakens labor laws and protections.^37^ Under the paradigm of labor market flexibility embedded in neoliberalism, Canadian governments implemented various policies aimed at rolling back the achievements of labor movements over the past century by hindering unionization and union activities, and by reducing or reinterpreting labor relations laws and regulations that support job security and other worker rights.^38^

The economic crisis of the 1970s marked the beginning of the government's erosion of free collective bargaining in Canada. During this period, the state increasingly used its power to end legal strikes through back-to-work legislation. This trend continued into the 1980s, as federal and provincial governments limited workers’ ability to strike. Governments weakened long-standing labor laws and regulations that included replacing card-check certification with mandatory elections for unionization, raising the thresholds needed for a successful union election, and excluding supervisory employees from collective bargaining units.^39^

Consequently, union membership declined from 38% in 1981 to 29% in 2022.^40^ Collective bargaining coverage declined in direct relation to losses in union membership.^41^ The restructuring of government programs for the unemployed in Canada was another policy shift reflective of neoliberal governance. In the mid-1970s, more than 80% of workers were eligible for unemployment insurance benefits. However, by 2012, fewer than 40% of workers met the stricter eligibility requirements. Additionally, for those who did qualify, employment insurance became less generous in terms of income replacement.^38^ These changes collectively weakened the power and influence as well as the economic and social security of working class Canadians. As noted by Thompson (2023):

Over one-third of Canadian workers hold precarious jobs, in which they face eroded worker protections and suppressed wages. These outcomes are not accidental but stem from deliberate policy decisions aimed at satisfying bosses’ drive to maximize profitability.^42^

### Housing

The Canada Mortgage and Housing Corporation concept of core housing need (CHN) identifies three aspects of problematic housing: inadequate (requires major repairs); unsuitable (not enough bedrooms for the size and composition of the household according to the National Occupancy Standard); or unaffordable (costs are more than 30% of a household's total before-tax income). The latest figures from 2021 indicate that 10.1% of Canadians experience CHN with figures ranging from 6.0% in Quebec to 32.9% in Nunavut. Of those in CHN, 77.1% live in unaffordable housing, 4.4% in inadequate housing and 5.5% in unsuitable housing with 13% of those in CHN experiencing more than one aspect. Across Canada, renters are much more likely (20% of renters) than homeowners (5.3% of owners) to experience CHN. The situation in Canada's largest cities is worse where the unaffordability rate is 33.2% for renters and 14.8% for homeowners. The rates for renters and homeowners in the four largest cities are, respectively, Toronto, 40.5% and 25.2%, Montreal, 27.9% and 19.6%, Vancouver, 38.5% and 25.4% and Calgary, 34.5% and 16.4%.^43^

As its most extreme manifestation, approximately 150 000 to 300 000 Canadians experience homelessness and access emergency shelter services in any given year. Given the difficulties connecting with those who are homeless—living/sleeping rough, couch-surfing, or staying in shelters—the numbers are likely much higher than these estimates suggest. In Toronto, Canada's largest city, over 10 000 people are homeless each night.^44^ As problematic as these figures are, they may not be reflecting current realities. A recent survey in Canada reports:

Today, 59% of Canadians—including 75% of renters—are sacrificing other basic needs such as food, clothing, living essentials, and education to afford rent or mortgage payments. Those sacrifices are taking a toll. Two-thirds of renters feel their mental health and well-being are impacted by their rent costs and one-third of homeowners say the same is true for the cost of their mortgage. The impacts are particularly evident for young Canadians, whose major life decisions, including family planning, where to live, and where to pursue a career are all at play.^45^

The evolution of the housing market illustrates the growing contradictions between capital accumulation and social reproduction. While most Canadians would likely now see the housing market as independent of government intervention, housing supply has historically been within the realm of government responsibility. From the late mid-1930s to 1993, the federal government (with cost-sharing from the provinces) funded construction of 650 000 housing units.^46^ The 1935 National Housing Program enabled new housing construction and repair and upgrading of existing homes and subsequent National Housing Acts of 1944 and 1954 built about 850 units per year throughout the country.^47^

Amendments to these Acts in 1964 created 200 000 new housing units over the next ten years such that prior to the 1980s, few Canadians were homeless. Although modest, these federal housing programs and the introduction of rent control in some provinces ensured affordable housing for many low- and middle-income households. By the 1970s with the growing dominance of neoliberal ideology, federal funding commitments were reduced, leading to a decline in the construction of new social housing. The mid-1990s saw the federal government—quickly followed by provincial governments—withdrawing from housing provision such that Canada by 1993 had terminated all federal funding for new social housing except on Native reserves.^48^

In 1999, under Bill C-66, the Canada Mortgage and Housing Corporation (CMHC) shifted its focus from providing affordable housing to the mortgage insurance business, transforming housing into a financial asset rather than a public good. As part of this shift, CMHC made it easier for lenders to offer mortgage loans by loosening insurance requirements. This change boosted housing demand and attracted financial capital into the profitable housing sector.^49^ During the same period, the affordability of existing affordable housing also came under threat through the process of financialization. In this way, housing has become the commodity of choice.^50^

Today, Canada has one of the most privatized and unaffordable housing sectors among OECD member countries with social housing making up only 3.5% of the total housing stock.^51^ Since 2005, the house price-to-income ratio in Canada has increased more sharply than in any other G7 country. By 2021, the index had reached 164.8, indicating that since 2005 home prices have grown 64.8% faster than household incomes since 2005.^52^

The federal government made a modest effort to re-engage in the provision of affordable housing, starting with the launch of programs such as the Affordable Housing Initiative (AHI) and the Investment in Affordable Housing (IAH) in 2001, which continued into the 2010s. In 2017, Canada introduced its first National Housing Strategy, which recognized access to adequate housing as a human right and aimed to increase the supply of social housing. However, these efforts did not reverse the significant reduction in government involvement that had occurred in previous decades. By 2017, federal funding for affordable housing was less than 20% of what had been allocated in the 1976 federal budget.^53^ In June 2024, the Liberal government announced a $1.5-billion co-op housing program (Gibson, 2024) that by March saw announcement of the first co-operative housing projects to receive funding under this program.^54^ It is unclear how many units will be built under this program. As noted by Moscrop:

When a half of the population is spending 50% or more of their household income on housing costs, the market is profoundly broken and we must reconsider the nature of its very existence as a hypercommodified space. Until we do so, we’ll continue to read headlines about desperate people seeking unworkable solutions to a crisis they’ve had no role in creating but whose effects they feel every single day of their lives.^55^

### Food

Household food insecurity (HFI) is when a household's financial ability to purchase sufficient nutritious food is limited due to lack of income associated with societal structures and policies.^56^ According to Statistics Canada, 2007 HFI rates of approximately 7.7% of households ballooned to 18.4% by 2022 indicating that roughly 6.9 million people, including nearly 1.8 million children, were living in households struggling to afford necessary food.^57,58^

Canada has faced food-related issues throughout its history due to droughts and economic depression. During these times, emergency charitable food delivery systems operated to address community food insecurity, but food banks, as we know them today, were virtually nonexistent. It was only in the 1980s coinciding with the rise of HFI that food banks emerged.^59^ The increase in HFI coincided with Canada's shift towards neoliberal inspired policies that reduced government support for meeting Canadians’ basic needs. Canadian welfare retrenchment was primarily implemented through significant cuts during the 1990s to key income security programs through the revision of the Canada Assistance Plan (CAP). Historically CAP required the federal government to cover 50% of provincial social program costs stimulating social spending and its replacement by the Canada Health and Social Transfer, which involved block grants, resulted in $7 billion in cuts to provincial funding for health, education, and welfare.^60^

By transferring much of the costs of social programs to provinces and territories who did not possess financial capacity to deliver these protections, along with federal cost-control and deficit reduction strategies, social expenditures were substantially reduced, leading to increased levels of HFI. Food banks and community food programs have also come to be seen as an extension of the principle of subsidiarity, where the government shifts its social responsibilities onto local communities and individuals.^61^

The relationship between employment conditions and HFI is apparent as a significant majority (78%) of food-insecure families depend primarily on paid employment for their income.^62,63^ In 2019, Canada established first national food policy, Food Policy for Canada (FPC) envisions a future where everyone in Canada has access to sufficient, safe, nutritious, and culturally diverse food.^64^ Approximately half of the $144.4 million in new funding targeted food insecurity through community-based programs that by focusing on local food production and distribution do not address broader issues of income distribution and employment conditions.^65^ Not surprisingly, FPC failed to reduce food insecurity in Canada as detailed above.^66^

Canadian governments' persistent focus on food banks, local food production, and the benevolence sector as primary solutions to household food insecurity denies the influence upon HFI of neoliberalism marked by fiscal austerity and reductions in welfare programs.^67^

### Healthcare

The establishment of Medicare in Canada is often interpreted from a critical social science perspective as a compromise between the government and the capitalist class, with the state balancing the needs of the working class and the interests of capitalists.^68^ The universal medical coverage in Canada was introduced in 1966 with a cost-sharing arrangement whereby the federal government covered 50% of the provincial health costs. In return, the provinces were required to meet four key criteria of comprehensiveness, portability, public administration, and universality.^69^ It was implemented in 1968, and by 1971, it ensured that all Canadians had access to prepaid essential medical services.^70^

Throughout the 1970s and 1980s, as neoliberalism gained influence, both federal and provincial governments sought to control spending, resulting in a series of legislative changes that shifted the financial responsibility of healthcare costs. In 1995, the traditional 50-50 cost-sharing model between the federal and provincial governments was replaced by a single, reduced block fund known as the Health and Social Transfer.^71^

After the establishment of block funding in the mid-1990s, the Canadian federal government continued to reduce its financial contributions to provincial spending.^72^ In 2002, the Romanow Commission on the Future of Healthcare in Canada revealed that federal cash transfers in the fiscal year 2001/02 covered only 18.7% of the provinces’ and territories’ expenditures on hospital and physician services.^73^

Since then, the federal government entered into a series of agreements with provincial and territorial governments (in 2000 and 2003) to renew the federal share of healthcare funding. Specifically, the Canada Health Transfer (CHT) was enhanced through a base adjustment and an annual six percent increase, known as the escalator, until the 2016/17 fiscal year. Starting in the 2017/18 fiscal year, the growth of the CHT would be tied to a three-year moving average of nominal GDP growth, with a guarantee that funding would increase by at least three percent each year.^74^

In the decades since the implementation of the new transfer system, Canadians have faced several significant challenges in accessing healthcare, including long wait times for specialist care, overcrowded emergency departments, and limited access to primary care providers.^75^ Statistics show that 18% of Canadians wait more than four months for elective, non-urgent surgery, while 30% wait over two months for specialist referrals.^76^ In 2024, over six million Canadians did not have consistent access to primary care physicians. Among those who did, one-third reported difficulties in booking appointments, contributing significantly to emergency room wait time crisis across the country.^77,78^

The Canadian healthcare system is expected to encounter increasing pressures in the near future due to rising service costs and an aging population. Projections indicate that by 2050, roughly one in four Canadians will be over the age of 65, compared to the current one in six. This demographic shift is anticipated to lead to a more than 21% increase in real per capita healthcare costs by 2050, compared to 2021 levels.^79^

These challenges within Canada's healthcare system have generated worries that Medicare is not effectively addressing the health needs of the population and is financially burdensome in its current state. Numerous solutions have been proposed, yet the neoliberal ideological commitment of Canadian governments has introduced growing complexity and uncertainty in shaping future healthcare policies and reforms. Some advocates propose increasing private financing of healthcare through out-of-pocket payments or private health insurance. Others suggest transferring more services to for-profit clinics.^80^

The idea of creating a parallel private healthcare system continues to be supported by some, despite considerable evidence showing that healthcare systems relying more on private financing generally perform worse when it comes to providing universal, equitable, accessible, and high-quality care, and do not necessarily result in better health outcomes. Privatization could lead to a system where access to healthcare is based more on an individual's ability and willingness to pay rather than need, thereby reducing equity in healthcare access. Allowing a parallel private system does nothing to address pressures in the public healthcare system. While those who can afford private care may experience improved outcomes, this benefit could be offset by worse outcomes for those who cannot afford to pay.^79,81–83^ In Ontario, Canada largest province, the Conservative government has been paying private for-profit health care providers over twice what would be the costs in public hospitals for cataract and knee arthroscopy. This is seen as an attempt to starve the public system and develop and alternative for-profit healthcare system. This appears to be happening all across Canada.^84^

Healthcare has always been a site for private sector capital accumulation through investment and growing commodification in Canada and other countries with public healthcare systems.^85^ Commodification through private for-profit investment undermines access to healthcare with special impact on the health of those in the lowest income groups.^86^ Healthcare is becoming commodified as governments shift away from the post-World War II commitment to provide healthcare towards neo-liberal inspired capital accumulation.^12,87^

## Analysis and Discussion

Critiques of social issues are often contentious in practice, primarily due to the difficulty in reaching consensus on what constitutes the problem. In the Canadian social context, the capitalist paradigm with its structures and processes, acts as the underlying causal mechanism shaping the material conditions of life independently of our awareness. Meanwhile, what is directly experienced by society, is the polycrisis affecting the living and working conditions of the people and threatening the public health care system and the health of Canadians.^17^

While many consider the current polycrisis as representing a convergence of unfortunate developments, there exists a long-standing critique that places ongoing crises—and polycrises—within the frame of the structures and processes of the capitalist economic system.^7,8,88–93^ For Marx, crises of capitalism are a normal aspect of its operation due to the separation of production from consumption.^94^ As explained by Meiville:

Just as they did under feudalism, existing economic relationships come into conflict with developing productive forces: economic crises ‘put on trial’ bourgeoise society. The great difference now is that due to the intense competition between capitalists, mass impoverishment and immiseration results, not from inadequate production, but from overproduction, a glut of products that go unsold. Bourgeois society responds in three main ways to this: with the destruction of productive forces, as, for example, in catastrophic slumps; by expanding into new markets, as in the global spread of imperialism; and/or with increased exploitation, where it's already entrenched. Which of course sets up more crises to come.^95(p47)^

Although these crises cause significant hardship, they do not destroy capitalism. Instead, Marx contended that such crises are necessary for capitalism's expansion, as they eliminate overproduction strains, drive out inefficient producers, and allow for a renewed balance between production and demand. However, Marx warned that this expansion would lead to increasingly severe crises over time. Ultimately, he believed that capitalism would be overthrown by the exploited workers, rather than collapsing due to an economic catastrophe. The reality of recurring crises under capitalism has been borne out. Carroll's and Sapinski's list of financial crises since World War 1 include 1929, 1937, 1947, 1951, 1953, 1957, 1960, 1974, 1980, 1981, 1990, and 2008.^96^

The vulnerability of the capitalist system in Canada to crises became evident during the Great Depression of the 1930s, which nearly led to the collapse of the global economy following the upheaval of World War I. Although post-World War II policies, particularly Keynesian strategies, seemed to stabilize capitalism for a quarter-century, this stability unraveled in the 1970s due to rising oil prices, increased costs, and strengthened labor unions demanding higher wages.^97^ This period paved the way for neoliberalism, which focused on reducing public spending and welfare but failed to resolve the escalating crises inherent in the capitalist system. Instead, neoliberalism contributed to another phase of economic turmoil. Several destabilizing forces emerged during this period, including increased international competition, the growth of debt, deregulation of financial markets, and the financialization of capital.^98^

The ongoing crises of capitalism have significantly impacted Canadian social policy, with profound implications for public health, particularly through the deterioration of the quality and equitable distribution of key SDOH. Since the rise of neoliberalism, Canada has undergone sustained welfare retrenchment, marked by cuts to government spending on social services. This reduction in spending has been further exacerbated by decreasing government revenues, largely due to various tax cuts influenced by capitalist ideologies.

In this social context, where the state provides fewer goods and services due to welfare retrenchment, income and wealth emerge as critical SDOH. Yet, during this period, economic conditions were marked by poor employment outcomes, stagnant wages, and a shift towards less stable jobs, driven largely by neoliberal anti-labor policies that prioritized market flexibility over worker security.^28^ The convergence of income stagnation and neoliberal-oriented policies in housing, food security, and healthcare over the past few decades has led to a polycrisis impacting the living and working conditions of many Canadians and resulting in a deterioration of the nation's overall health status.^99^

History serves both as a reference point and a potential guide for navigating future challenges. Throughout history, crises of the capitalist system have been inevitable, yet the impact of these crises has often been mitigated through government interventions, which have helped to shorten their duration or reduce their severity. Gourevitch categorized government responses to the crises of the past century into five distinct approaches.^100^ These strategies include socialization of ownership and planning, linked to socialist and communist approaches, which was notably employed in the Soviet Union and Eastern Europe during the Great Depression to mitigate severe economic inequality. Protectionism and Keynesianism, which were extensively implemented in the United States and Western Europe after the Great Depression and during the post-World War II recovery to boost economic activity through government spending and to protect domestic production. Neoclassical liberalism, which emphasizes market primacy, gained prominence in the United States and the United Kingdom during the 1980s following the stagflation crisis of the 1970s. Mercantilism, where the state supports specific sectors or firms, has often been seen in countries like China and Japan during economic restructuring efforts.

Nonetheless, the ongoing challenges and the convergence of multiple intertwined crises in the early twenty-first century raise questions about the effectiveness of past strategies in addressing the current polycrisis. Generally, three alternatives are considered viable. The first approach, often supported by neoliberals, advocates a return to normal approach, aiming to re-establish the pre-crisis status quo. The three primary components of this return to normal are fiscal austerity, the privatization and marketization of the public sector through public-private partnerships, and increased labor market flexibility. Yet, these measures will further shift the burden of resolving the crisis onto the working class, affecting them as taxpayers, recipients of diminished public services, or workers facing precarious employment and reduced incomes.^28^

The second approach calls for significant reform of the neoliberal model, acknowledging that a return to the previous status quo is unfeasible and that the interconnected nature of multiple crises requires a transition to adaptive strategies. This perspective is often viewed as an attempt to reimagine Keynesian approaches for the twenty-first-century polycrisis. It suggests that the government should play a more active role in the economy and consider public services as investments rather than liabilities. Proposals such as Universal Basic Income or Job Guarantee programs, increased taxes on wealth and excess profits, and reforms in the healthcare sector, especially re-examining the role of profit in areas like long-term care are already being discussed.^1^ However, it is believed that the concept of a polycrisis refers to a pivotal moment requiring a significant change in direction. Such reforms may, at best, only delay an inevitable radical shift rather than prevent it altogether.^7^

The third approach to addressing a polycrisis involves fundamentally transforming the capitalist system, advocating for a shift towards a socialist model where the economy is planned in the public interest. This perspective sees modest reforms as transitional steps toward more radical change. It emphasizes the need for nationalization of banks and financial institutions and calls for empowering trade unions to protect workers’ rights and improve labor conditions. Furthermore, it focuses on enhancing national capacity to meet domestic needs, reducing reliance on global markets.^1^ Yet, this transformation necessitates the mobilization of social organizations and movements that are unwilling to accept initial reforms as the ultimate solution. Currently, there is no major political party in Canada that explicitly identifies the inherent problems of capitalism or advocates for a transition to a post-capitalist socialist state.^101^

In Canada, the primary political expression of socialist thought was the Cooperative Commonwealth Federation (CCF). Founded in 1932, the CCF was an explicitly socialist political party whose original manifesto boldly declared its commitment to eradicating capitalism and implementing a comprehensive program of socialized planning. However, in 1956 the party's manifesto was replaced with the Winnipeg Declaration, which omitted any reference to replacing capitalism and downplayed the urgency of public ownership of the means of production. In 1961, the CCF was renamed the New Democratic Party (NDP) in response to electoral stagnation and the desire to appeal to a broader voter base. Since its formation, the NDP has moved further away from its socialist and social democratic origins, gradually transforming into a reformist capitalist party, reducing its emphasis on challenging the capitalist system or advocating for radical changes. As a result, the party's agenda now largely lacks any focus on mobilizing working-class power to confront capitalism directly.^101^

The core argument of this paper is that what is truly needed in Canada is a true new left-wing party that is genuinely committed to a post-capitalist social system. This party should focus on addressing the living and working conditions of working-class Canadians and should not accept reforms as final solutions but rather as steps towards a larger transformation. Yet, to effectively influence the predominantly neoliberal policy landscape in Canada, such a party would need the implementation of proportional representation in Canada's electoral process. Proportional representation would allow for a more accurate reflection of voters’ preferences and could enable a new left-wing party to gain representation in government.^102^ A 2019 Forum poll found that 58% of Canadians held positive views toward socialism, suggesting that Canadians might be open now to embracing systemic changes that align with post-capitalist socialist principles.^103^

## Conclusion

The root cause of the polycrisis in the working and living conditions of Canadians is the underlying capitalist system, particularly its recent iteration driven by neoliberal ideology.^104^ Capitalism, by its very nature, is an unjust economic system that inherently subjects the majority of the population to economic and social insecurity. The systemic issues inherent in capitalism indicate that to truly break the cycle where one crisis leads to another or worsens existing problems, it is necessary to dismantle capitalist social relations and establish an economic system based on socialist principles.

This transformation would involve profound structural changes, moving towards a new social structure that ensures everyone can lead decent and fulfilling lives. Such a change, however, would require a larger and more robust state, characterized by enhanced democratic principles based on new representational criteria. Institutions, such as a new left-wing party, could then provide greater influence for workers and other currently underrepresented sectors of society. A new left with a genuine commitment to the traditional manifesto of putting into operation the full program of socialized ownership appears necessary.
